# The effect of blended task-oriented flipped classroom on the core competencies of undergraduate nursing students: a quasi-experimental study

**DOI:** 10.1186/s12912-022-01080-0

**Published:** 2023-01-10

**Authors:** Li Ke, Lanlan Xu, Li Sun, Juan Xiao, Lingxuan Tao, Yixue Luo, Qiongya Cao, Yan Li

**Affiliations:** School of Nursing, Hubei University of Medicine, Shiyan, Hubei China

**Keywords:** Fundamentals of Nursing course, Task-oriented, Flipped classroom, Self-directed learning ability, Problem-solving skills, Critical thinking ability

## Abstract

**Background:**

The flipped classroom (FC) method is becoming increasingly popular in China's nursing education. It is an important breakthrough improvement in the quality of learning in nursing education reforms.

**Purpose:**

This study aimed to determine the effects of blended task-oriented flipped classroom (TFC) on nursing students undertaking the Fundamentals of Nursing course.

**Methods:**

A pre-and post-test quasi-experimental design was adopted. This study was conducted in the Autumn semester, 2021 academic year in a Chinese university. Using cluster sampling technique, this study enrolled second-year undergraduate nursing students from six classess who were studying Fundamentals of Nursing course. A blended TFC was developed and implemented with three classes (experimental group: *n* = 152). In-class traditional lectures were applied to the other three classes (control group: *n* = 151). The Self-Directed Learning Instrument, Problem-Solving Inventory, and California Critical Thinking Disposition Inventory were used to evaluate students’ learning outcomes, and final examinations were conducted at the end of after course. In addition, students in the flipped classroom group were required to answer five open-ended questions concerning their flipped classroom learning experiences.

**Results:**

Students in the experimental group showed significant improvement in academic performance compared to those in the control group (*p* = 0.001). Considering total scale and factors, students in the experimental grouped recorded significantly higher scores in self-directed learning ability, problem-solving skills, and critical thinking ability compared to those in the control group (*p* < 0.05). Furthermore, improved abilities and skills such as team cooperation, communication, presentation, identifying /solving clinical problems, and accountability were reported.

**Conclusion:**

A blended TFC teaching approach positively impacted students' core competencies and improved learning outcomes in the Fundamentals of Nursing course.

**Supplementary Information:**

The online version contains supplementary material available at 10.1186/s12912-022-01080-0.

## Introduction

There is a huge gap between nursing education and nursing practice [1]. Traditional lecture-based education is inadequate in enabling students to develop higher level thinking abilities such as critical thinking and problem solving [2, 3]. However, in clinical settings, nurses are expected to be highly competent and solve problems critically to meet the vast needs of patients and the population. Thus, it is essential for undergraduate nursing education to develop professional talents in the core competencies needed to deliver quality care in today's complex health care system. This highlights the need for students to improve their problem-solving and communication skills, team engagements and collaborations, critical analysis of evidence, consistent systems-based practice, self-evaluation, and life-long learning [4, 5]. Therefore, nursing educational reforms, especially teaching methods, has always been one of the main objectives of nursing educators in recent years in order to respond rapidly to the changes in the healthcare sector [6–8].

The flipped classroom (FC) model is an important development in improving the quality of teaching in nursing education and is increasingly becoming popular in nursing education in China [6, 9, 10]. The FC is a teaching strategy that promotes critical thinking, and ensures the smooth application of the knowledge acquired outside the classroom to real-world situations and problems [11]. In FC, active learning methods, and student-centered approach to learning are used [12]. Students voluntarily and actively obtain lecture materials before class via different means, such as reading or lecture videos, and then resolve problems during class time through strategies such as discussion, cooperation and debates [13]. The teacher's role has changed from a simple passer of knowledge to a leader and organizer of interactive pedagogical classroom activities [10]. Teachers can use games, experiments, group discussions, fora and diversified activities to guide students to apply and internalized their acquired knowledge and deepen their creativity, inquiry ability and teamwork spirit [14].

Studies have consistently found that the use of FC approach in nursing education presents some advantages over traditional lecture-based learning. It was found that the FC model improve academic performance [6, 11, 12], increase student satisfaction [15], and increase quality of teaching [15]. Nursing students found FC approach learning atmosphere more enjoyable [15]. The use of FC has also been reported to positively impact student's self-evaluated core competencies, including learning motivation [16, 17], critical thinking disposition [2, 6, 7, 11], self-rated learning ability [14, 17], self-directed learning ability [18], self-efficacy in practice [19], and problem-solving [14]. Accordingly, the FC method has been widely applied in various nursing education courses, including pharmacology, anatomy and physiology, health nursing, community health, et al. [15].

However, studies investigating the effect of flipped learning on the Fundamentals of Nursing course are scarce [4]. The Fundamentals of Nursing course is crucial for the training of nurses. It is a mandatory introductory course for nursing students learning clinical specialties such as internal medicine nursing, surgical nursing, obstetrics and gynecology nursing and pediatric nursing. Besides, it provides the necessary basic knowledge and skills for clinical nursing. Meanwhile, online classrooms are becoming increasingly popular, particularly, in light of changes in the teaching arrangements due to the ongoing pandemic [20]. Educators have developed a blended teaching model based on FC [6, 10]. However, to the best of our knowledge, studies on the design and practice of online and offline blended teaching based on the concept of FCs are uncommon. Moreover, most studies on the FC approach in nursing education were conducted in the United States or Europe [15]. Studies exploring the impact of the FC approach in China are still inadequate.

We, therefore, attempted to develop a blended Task-oriented flipped classroom (TFC) approach in the Fundamentals of Nursing course. In November 2020, the blended TFC for the Fundamentals of Nursing course was recognized as part of the first batch of the national first-class online and offline blended curriculum. This is one of the eight online and offline blended first-class courses relating to nursing majors in China [21]. TFC divides the learning objectives into small tasks and sets up task-oriented teaching activities to guide students to consolidate, internalize and apply knowledge and improve teaching effects. TFC was designed to help undergraduate nursing students reach advanced critical thinking and analytical competencies, evidence-based practice, problem-solving skills, communication and informatics, decision-making and clinical judgment, teamwork and collaboration, and life-long self-directed learning.

The theoretical basis that guided the development of TFC was constructivism [22]. Constructivism is a theory that emphasizes on the significant role of collaboration in learning. In this regard, learning becomes an active process in which learners co-construct meaning and understanding by applying concepts using various communicative methods and then share their new knowledge with peers [22]. Constructivism helps in nursing pedagogy by improving the ability to gather, analyze, and evaluate information experientially resulting in the development of a new framework [22]. While the content and requirements for nursing science continue to change and increase, nurses who are equipped to think critically become self-directed learners, and identify/solve clinical problems more effectively during patient care than those who memorize data without exploring the context of learned information.

In summary, there is an increase in the number of studies on flipped classroom learning in recent years with some promising results. Nevertheless, there is the need for more studies, especially using an experimental design with simultaneous intervention and comparison groups among nursing students in different educational contexts/content areas. Herein, we present a blended teaching model process based on TFC and demonstrates the impact of flipped learning approach particularly, the improvement in students' core competencies in the Fundamentals of Nursing course with the aim to provide the basis for nursing teaching reforms in China.

The hypotheses were:Students in the TFC course compared with those in the traditional learning classroom will display significantly improvement in self-directed learning ability, problem-solving skills and critical thinking ability. Students in the TFC will have better academic performance compared to students in the traditional learning classroom.

## Methods

### Study design and sample

**Fig. 1:**
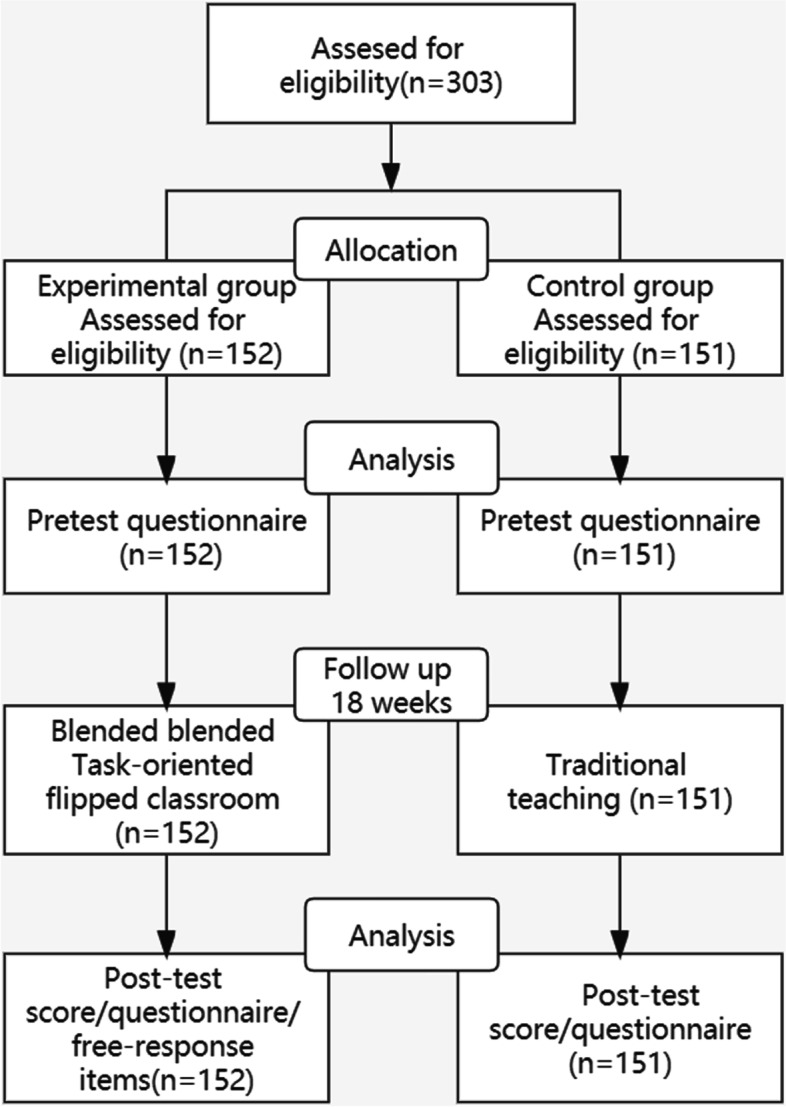
Participants flow diagram

A pre-and post-test quasi-experimental design was adopted. This study was conducted in the Autumn semester of 2021 in a Chinese university. Cluster sampling method was used. The sample comprised of all 303 second-year undergraduate nursing students studying Fundamentals of Nursing course in the third semester. These students were randomly divided into six 50- or 51-student parallel classes when they entered university and attended separate theoretical training classes. Thus, randomisation in this study was done at the class level. These six classes were randomly allocated to either an experimental group (three classes, *n* = 152) and a control group (three classes, *n* = 151) by coin flipping. The experimental group engaged in blended task-oriented flipped classroom, whereas the control group received traditional teaching. Figure 1 provides a flowchart of participants’ enrolment in the study.

### Teaching arrangements

The Fundamentals of Nursing is a mandatory three-credit hour course for second-year nursing students. The total number of hours required to complete the whole course is 57 h. The course instructors are faculty members with Ph.D and/ or Master degree in nursing (5 Associate Professors and 4 Lecturers). Each of them have at least eight years of teaching experience. Our team screened and integrated the course contents. We divided the course contents into nine chapters (Table 1). Each chapter consisted of 3 or 4 sections. Teachers, textbooks and contents were same for both groups.

**Table 1:** Teaching contents and credit hours (57)

chapter	contents	credit hours
0	Introduction	2
1	Patient admission and discharge care	5
2	Patient hygiene, rest and activities	7
3	Prevention and control of nosocomial infection	7
4	Observation of vital signs and hot and cold application for nursing	7
5	Diet and nutrient excretion	5
6	Administration of medication	8
7	Intravenous infusion and blood transfusion	6
8	Observation of disease condition and rescue of critical patients	5
9	Hospice care and nursing documentation	5

All nine teachers participated in faculty training in online self-directed learning and face-to-face in-class activities. Collective lesson preparation was conducted before teaching to ensure consistency in content and teaching materials. Teaching materials for FC, including lesson plan, coursework, learning task list, clinical cases, discussion topic, group report theme, group work and extensive learning materials were discussed and decided on collectively during lesson preparation by the faculty in charge of teaching this course.

### Procedure

#### Experimental group

**Fig. 2:**
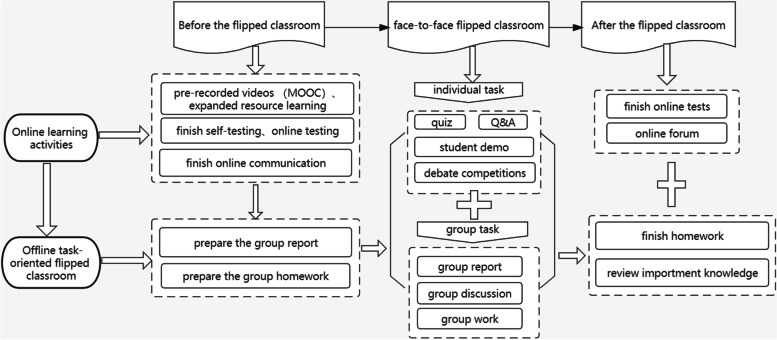
The Task-Oriented Flipped Classroom teaching design for The Fundamentals of Nursing course

In this study, the blended TFC incorporated online self-directed learning (via the "Learning Platform and MOOC") and offline face-to-face in-class activity. The model composed of three class components: before, during, and after classroom sessions. Figure 2 presents steps involved in the process.

Before FC, based on the number of participants in small offline classes, online virtual classes were created on the Learning Platform. The teacher provided learning task lists to students via the Learning Platform a week prior to the FC. Take chapter2, Sect. 3 an example (Appendix 1). Each section contains a special learning task lists which comprise of learning goals, contents, preparation materials, group report topics, group homeworks and other learning materials. All students log into the Learning Platform and register on the MOOC website to undertake self-directed learning [23]. The MOOC was designed and pre-recorded by our team with the help of a professional company. We included 234 mini-lectures videos that ranged from 3 to 20 min long, totaling 26 h. These videos were available online for free during the entire semester, and students could learn individually at their own pace. Online learning activities included pre-recorded videos, and expanded learning resources (expanded videos, relevant literature, etc.). In addition, self-testing (exercises on knowledge points and chapter tests), online communication, and online testing were included. Besides individual preparations, students in each group were required to use materials, prepare group report slides, discuss homework provided by the teacher, and present a report.

During the face-to-face TFC process, subclasses were formed which consisted of 25 ~ 26 students. This was further divided into eight learning groups (3 ~ 4 people/group). Classroom tasks included group work (reports, discussions, homework), and individual tasks (quizzess, Q&A, demonstrations, debates). All students were asked to complete group work together as a unit, and then complete individual tasks differently. Students were allowed to discuss cases and questions provided by the teacher. A representative from each subgroup was asked to summarize the presentation, while the remaining students provided additional answers. The teacher then commented on each summarized report, and guided students on how to think and discuss. Q&As included picture/video error findings and matching. Students also demonstrated their individual skills and debated controversial topics. Students were required to answer a 10-question online quiz to assess their knowledge on the content at the beginning and end of the FC. Afterwards, teachers provided explanations based on their answers.

After the FC, students were asked to submit homework, raise questions on online platform and review teaching videos. All study materials were recorded via the platform and could be accessed by all course instructors. Teachers communicated, discussed, answered difficult questions, and issued classroom evaluation questionnaires to students during the process.

#### Control group

The control group underwent face-to-face traditional classroom teaching. The course consisted of lecture-based learning(LBL); this is where the instructor uses PowerPoint presentation slides for each topic. Teachers arranged their instructions according to the curriculum, and students focused on what they have been taught. There were no extra class activities or interventions provided.

### Measures

After receiving a verbal briefing on the research and completion of the informed consent form, both student groups (experimental group and control group) completed the same data forms and scales. Data collected included demographics, course exam scores, learning outcomes and students' experiences. The demographic questionnaire included students' age, gender, last academic year GPA (the highest possible GPA is five). One research assistant in each class was invited to assist in participant’s questionnaire distribution. Pre-test data were collected one week before the course, while post-test data were collected one week post course completion.

#### Academic performance

The course assessment contained two parts: final exam (70%) and process assessment (30%). The final exam was conducted one week after course completion.

The total score of the final exam was 100, consisting of 45 multiple-choice (45%), 10 true or false questions (10%), 5 noun explanations (15%), 5 short answer questions (20%) and 1 case analysis question (10%). These examination questions were taken from the question bank of the Fundamentals of Nursing course according to the teaching outline. They were reviewed by two other lecturers and approved by the teaching team lead. The content validity of the final examination was 0.9, as analyzed by two reviewing lecturers. The average difficulty coefficient and discrimination of this paper were 0.64 and 0.24, respectively. The structural validity, reliability coefficient and reliability of examination questions were 0.17,0.81, and 0.78, respectively.

The process assessment of the experimental group included group reports(10%), group discussion(5%), group homework(5%), individual tasks(5%), and online learning progress (5%). The scores of group tasks and individual tasks in the experimental group were recorded in class by teachers using special record forms. Online learning of the experimental group were recorded and assessed automatically on the learning platform. The process assessment of the control group included Students’ performance (20%), and individual homework(10%). Homeworks were mainly marked by teachers using same criteria. Students’ performances were assessed based on their response during offline classes.

#### Students’ self-directed learning ability, problem-solving skills, and critical thinking ability

To understand TFC learning outcomes, particularly, regarding students’ mental self-evaluation processes, the following instruments were used.

##### Self-Directed Learning Instrument (SDLI)

The Self-Directed Learning Instrument (SDLI) measures self-directed learning ability among nursing students, developed by Cheng S F et al. in 2010 [24]. It contains 20 items in total with 4 subscales that measure the following dispositions: Motivation for learning (6 items), Planning and implementation (6 items), Self-monitoring (4 items), Interpersonal communication (4 items). Items are scored on a 5-point Likert scale: 1 = strongly disagree to 5 = strongly agree, with higher scores indicating higher trends of self-directed learning (total scores range from 20 to 100 points). The internal reliability coefficients in our study were 0.87 (pre-test) and 0.89 (post-test).

##### Problem-Solving Inventory(PSI)

Students’ problem-solving skills were measured by The Problem-Solving Inventory(PSI) developed by Andrew M.H.Siu et al. in 2005 [25]. The translated and modified Chinese version was more suitable for Chinese students [26]. The Scale measures individual problem-solving skills and deficiencies, which composed of 25 questions with five dimensions- Positive Problem Orientation (PPO, 3 items), Rational Problem Solving (RPS, 7 items), Negative Problem Orientation(NPO 5 items), Impulsivity\Carelessness Style (ICS, 5 items), and Avoidance Style (AS, 5 items). Each question was answered using a 5-point Likert-type scale ranging from 1(least matched) to 5 (highly matched), and higher scores of the first two domains indicated greater problem-solving ability (total scores ranged from 25 to 125 points). The internal reliability coefficients in our study were 0.86 (pre-test) and 0.87 (post-test), and the values for the subscales ranged from 0.79 to 0.86.

##### California Critical Thinking Disposition Inventory (CTDI)

The California Critical Thinking Disposition Inventory (CTDI) developed by Facione et al. in 1994, assessed critical thinking ability among nursing students in different cultural contexts [27]. Chinese researchers translated and modified the CTDI to make it more suitable for Chinese students [28]. There are 70 items in total and consists of 7 subscales: truth-seeking, open-mindedness, analyticity, systematicity, critical thinking, self-confidence, inquisitiveness, and cognitive maturity. Each item was scored using a 6-point Likert-type scale ranging from 1 (strongly disagree) to 6 (strongly agree), with higher scores indicating higher trends of critical thinking ability (total scores range from 70 to 420 points). The internal reliability coefficients in our study were 0.85 (pre-test) and 0.88 (post-test), and the values for the subscales ranged from 0.78 to 0.86.

#### Students’experience

Finally, student' perceptions about TFC were collected after TFC experience. Five free-response items facilitated discussions on their general perceptions of the approach to teaching the Fundamentals of Nursing course, and how this approach facilitated or hindered learning: (1) how did you prepare before in-class activities and what difficulties did you face during the preparation? (2) What are your thoughts on teamwork concerning TFC? (3) In your opinion, which comprehensive abilities have improved with TFC? (4)How did you experience the TFC ? (5) What recommendations can you give to improve the learning of this topic?

### Data analyses

Data were analyzed using the IBM SPSS 25.0 statistical package. Descriptive statistics (percentages and ranges) analyzed students' demographic characteristics.

To compare students' level of Self-Directed Learning, Problem-solving, Critical thinking before and after the implementation of flipped learning, the paired sample t-test and independent-sample t-test were applied. To measure differences in students' scores between groups, independent t-tests were used. Students' responses to the above-mentioned five open-ended questions were also analyzed by thematic analysis.

## Results

### Characteristics of participants

Among the 303 nursing students who participated in the study, 274(90.4%) were females, of whom 132 were in the experimental group, and 142 in the control group. Twenty-nine (9.6%) students were males (experimental group = 20, control group = 9). Students were 19.88 years and 19.80 years old on average in the experimental and control groups, respectively. There was no statistically significant difference between the experimental group compared with control group in terms of age, gender and last-academic-year GPA (*P* > 0.05). Characteristics of participants are shown in Table 2.

**Table 2:** Characteristics of participants

Characteristics	Experimental group (*n* = 152)		Control group (*n* = 151)		*P* value
	Mean	SD	Mean	SD	
Age	19.88	0.75	19.80	0.70	0.624^a^
Last- academic-year GPA	3.45	3.33	3.39	3.03	0.115^a^
Gender	N	%	N	%	0.538^b^
Male	20	13.2	9	6.0	
Female	132	86.8	142	94.0	

^a^ The independent-sample *t*-test; ^b^ The chi-square test

### Academic performance of participants

The academic performance (82.18 ± 8.97) among students in the experimental group was statistically significantly higher than those in the control group (77.95 ± 11.76) (*p* = 0.001).

### Quantitative data: self-directed learning ability, problem-solving skills, critical thinking ability

There was no statistically significant difference between the experimental and control groups regarding pre-test SDLI, PSI, and CTDI: all domains and total scores. Comparing experimental and control groups' scores regarding post-test SDLI, PSI, and CTDI, there were statistically significant differences in total and in most domains (all *p*-values < 0.05), excluding NPO of PSI, and systematicity of CTDI. Within groups, there were statistically significant improvements in the experimental group pre/post-test for all domains and total SDLI, PSI and CTDI (all *p*-values < 0.001), excluding NPO of PSI, and systematicity of CTDI, while there were non-significant results in the control group (Table 3).

**Table 3:** Comparison of scores of Self-Directed Learning ability, Problem-solving skills, and critical thinking ability before and after between experimental and control groups, mean (SD)

Variables	Control group		Within the group	Experimental group		Within the group	between groups(Pre-test)	between groups(Post-test)
	Pre-test	Post-test	*p*-Value^a^	Pre-test	Post-test	*p*-Value^a^	*p*-Value^b^	*p*-Value^b^
**SDLI**								
Motivation of learning	20.45 ± 3.64	20.48 ± 2.94	0.927	20.76 ± 4.07	23.54 ± 3.89	*p* < 0.001	0.491	*p* < 0.001
Planning and implementation	19.50 ± 3.32	19.83 ± 2.71	0.255	19.89 ± 4.16	22.76 ± 4.39	*p* < 0.001	0.374	*p* < 0.001
Self-monitoring	12.95 ± 3.32	13.27 ± 1.97	0.117	12.75 ± 2.79	15.09 ± 2.95	*p* < 0.001	0.483	*p* < 0.001
Interpersonal communication	13.54 ± 2.30	13.84 ± 2.07	0.175	14.01 ± 2.74	15.16 ± 3.03	*p* < 0.001	0.112	*p* < 0.001
Total	66.45 ± 9.24	67.42 ± 7.47	0.195	67.40 ± 12.45	76.54 ± 13.12	*p* < 0.001	0.451	*p* < 0.001
**PSI**								
Positive Problem Orientation	16.90 ± 2.67	17.03 ± 2.42	0.551	16.87 ± 3.02	17.65 ± 3.12	0.032	0.922	0.013
Rational Problem Solving	11.46 ± 3.38	12.05 ± 3.41	0.055	11.60 ± 3.45	13.44 ± 4.31	*p* < 0.001	0.731	*p* < 0.001
Negative Problem Orientation	16.16 ± 2.16	16.14 ± 2.53	0.926	16.22 ± 2.34	15.20 ± 2.80	*p* < 0.001	0.798	0.837
Impulsivity\Carelessness Style	11.94 ± 2.10	11.60 ± 2.25	0.082	11.64 ± 2.23	11.05 ± 2.17	0.002	0.236	0.032
Avoidance Style	19.80 ± 3.21	19.32 ± 2.87	0.076	19.89 ± 3.24	18.45 ± 3.45	0.001	0.801	0.048
**CTDI**								
truth-seeking	36.28 ± 6.81	36.57 ± 5.25	0.172	36.96 ± 7.81	38.43 ± 6.17	0.027	0.423	0.005
open-mindedness	39.39 ± 5.37	39.53 ± 4.15	0.467	39.92 ± 6.41	42.36 ± 6.31	*p* < 0.001	0.435	*p* < 0.001
analyticity	39.82 ± 4.47	40.05 ± 4.46	0.115	39.72 ± 6.08	41.30 ± 5.17	0.002	0.865	0.025
systematicity	37.19 ± 5.82	37.01 ± 4.99	0.175	37.34 ± 5.12	37.44 ± 6.12	0.823	0.829	0.507
critical thinking self-confidence	38.44 ± 6.36	38.67 ± 6.49	0.121	38.099 ± 7.15	40.24 ± 6.45	*p* < 0.001	0.658	0.035
inquisitiveness	39.85 ± 6.92	40.21 ± 5.49	0.085	40.28 ± 7.28	41.89 ± 6.97	0.008	0.600	0.020
cognitive maturity	39.18 ± 7.88	39.68 ± 5.60	0.089	40.88 ± 8.80	44.72 ± 7.69	*p* < 0.001	0.077	*p* < 0.001
Total	270.15 ± 27.26	271.72 ± 20.92	0.079	273.18 ± 29.20	286.39 ± 27.33	*p* < 0.001	0.351	*p* < 0.001

^a^ The paired sample *t*-test; ^b^ The independent-sample *t*-test

### Nursing students' experiences in the TFC

Regarding free-response questions, most students (71.4%) found the pre-class FC activity "efficient", whereas 14.3% had a poor experience. "Too much preparation" and "laziness" were immediate causes of poor experience. As for teamwork in class, almost all students were satisfied. They reported that teamwork helped them to increase their sense of belonging. With respect to improving comprehensive ability after TFC, students felt they had improved their abilities in many respects, including team cooperation, communication, presentation, identifying /solving clinical problems and accountability. When asked about their TFC experience, most students said it was nice, interesting, multivariant, and attractive. Regarding areas of improvement, one student noted that too much preparation for pre-class activity made self-studying burdensome. Some students rushed to answer questions too often, resulting in fewer opportunities for other students, such as introvertes.

## Discussion

To the best of our knowledge, our study is one of the first to investigate the effects of TFC on learning the Fundamentals of Nursing course. Our study showed that TFC teaching approach could create positive learning outcomes, including improvement in academic performance, improvement in student's self-directed learning ability, problem-solving, and critical thinking skills. Moreover, students’ competencies, e.g., teamwork, communication, presentation, identifying/solving clinical problems and accountability were reportedly improved. Our study confirmed the effectiveness of TFC in the learning of Fundamentals of Nursing course. This can be partly explained by the task-based FC's teaching design of the nursing course.

The results of this study showed that academic performance of students in the experimental group was better than those of the control group (*p* = 0.001). This result is consistent with studies indicating that student academic performance could be improved by using the FC method [6, 29, 30]. This might be partly explained by the teaching design of the Fundamentals of Nursing course, which is the first nursing course with blended TFC learning at our university. The blended TFC learning was applied during both group and individual learning activities. This can effectively improve students' classroom participation. More importantly, this encouraged voluntary supervision among group members, as students took decisions together- both stronger and weaker ones. In this process, students get more attention, affirmation and motivation from teachers, which is also conducive for stimulating and activating students' participation in the classroom and arousing students' intrinsic motivation for learning.

In our study, participants' self-directed learning ability, problem-solving and critical thinking skills significantly improved after taking the TFC. The observed progress was consistent with previous studies [2, 6, 11, 14, 16–18]. The rapidly changing health care environment expects nurse practitioners to have the ability to continuously improve patient care based on constant self-evaluation and life-long learning. In nursing, students enter the field with extensive professional training and constantly update their knowledge through self-directed learning [24]. Herein, we created a student-centered learning environment to help them take on learning responsibilities and become autonomous. All students were required to undertake self-directed learning according to learning task lists before the FC. They had to spend more time on online learning activities. In the meantime, students had to exibit teamwork skills to complete tasks such as group reports. It was difficult to participate in offline TFC if pre-class activities were not well prepared. Participants were both under pressure and motivated to prepare and/or present in class, which helped develop into mature students capable of self-learning and improve their learning motivation and self-monitoring. Additionally, given that FC allows learners to be independent and move at their own pace, students who make pre-class preparations with FC can progress at their own learning speed [31].

The results showed significant difference in increased problem-solving skills in the experimental class compared with the control class. FC can help nursing students to accurately grasp diverse and complicated scenarios and respond creatively to foster superb decision-making and problem-solving skills [32, 33]. In this study, all group tasks and individual tasks in the TFC originated from real clinical cases. Coupled with teachers' systematic and earnest instructions, students could engage in in-depth thinking and step-by-step discussions, providing crucial insights into clinical case analysis and problem-solving [6]. This positively affects students' ability to solve problems in a strategic, systematic and integrated manner. They become more pro-active, objective and calm when solving problems, instead of being passive and one-sided. Students also reported their inability to apply integrative thinking to solve problems by analyzing cases together. They would try various ways to solve group and individual tasks, such as revisiting contents learned from anatomy pathophysiology classes and reading literature. These activities enriched their learning experience and were helpful to improve problem-solving. This flipped-classroom model is vital in preparing them to meet future challenges in healthcare; they need to be creative problem-solvers [14, 16].

Additionally, the offline face-to-face TFC enabled participants to learn in a collaborative and student-centered active learning environment. Every student was encouraged to contribute in the team, effectively present one's own opinions, take turns to report, and critically think and answer questions from others [32]. This offered more opportunities to students to present themselves, and promoted voluntary supervision among group members, as students made decisions together. After the FC, students felt refined in their core competencies variously such as in critical thinking, teamwork capabilities, communication, functional performance, presentation, and accountability. As shown in our results, TFC approach can prepare students for life-long learning and give them skills needed to meet future challenges in the profession.

Nevertheless, some students reported improvements in some areas, for instance, too much time was spent on pre-class preparation. Students in another study who had experienced FC reported that watching recorded lectures and studying for quizzes were time-consuming [29]. Indeed, lengthy lectures can be boring and tiring for students. After our study, we considered shortening the length of the pedagogical videos. Indeed, students could stop and re-play each video at any preferred time. Furthermore, some students rushed to answer questions too frequently, resulting in fewer opportunities for other students. Indeed, more opportunities should be given to introverted students.

This study had several advantages. First, we created different tasks with problems to be solved which required teamwork. Learning objectives were incorporated into various specific tasks; as students actively engaged in new tasks, they were much more prepared and engaged in unfamiliar discussions. Second, in some FCs, only a portion of class hours were flipped. This was not sufficient to fully foster the development of high analytical and critical thinking skills in nursing students. Our FC was conducted throughout the semester to identify and assist weak students. This approach was used in all aspects of the course to ensure that students can develop a pattern and way of learning that is not disrupted when a different approach is employed.

The study had several limitations. First, the study was carried out in a single semester with a single course, limiting the validity and reliability of the findings, especially, improvement in students' core competencies. Differences in students' motivation and preparation approaches existed. Thus, more studies assessing the effectiveness of blended task-oriented FC learning for other nursing subjects are needed to validate our findings [34]. Second, our study only measured short-term outcomes in students' perception of learning experience rather than changes in their learning habits and clinical performances. Future studies on continuous evaluation of students’ performance in clinical practice is warranted. Third, our qualitative data was obtained from free-response questions. Conducting face-to-face interviews with individuals or groups of students after the end of the semester might have been a better option. However, we chose free-response questions over other options to minimize students' burden during the school term and avoid recall bias, particularly after students have obtained their final grade for the course. Lastly, the study data included self-reports with no blinding procedure. Also, both groups studied same program in the same institution, cross-contamination might have occurred. In addition, students in both groups could have communicated or shared their learning strategies, which might have influenced the results. Thus, the application of a randomized clinical trial study design and rigorous control for heterogeneity factors during the intervention period may confirm the flipped classroom’s probable effects.

## Conclusion

This study showed that blended TFC method could be an effective pedagogical strategy for learning Fundamentals of Nursing course. Students in this program were satisfied with this method and demonstrated improvements in students' academic performance, self-directed learning ability, problem-solving, and critical thinking skills. Moreover, this approach inspired students to learn actively and more efficiently and improved other core competencies such as team cooperation, communication, presentation, identifying /solving clinical problems, and accountability, which are essential elements for professional healthcare practice. Herein, we showed that TFC approach is one of the most effective teaching methods for today's complex revolution in nursing curricula and may enhance nursing students' core competencies to address numerous challenges.

## Supplementary Information

**Additional file 1.**

## Data Availability

These data during the current study are not publicly available due to confidentiality but are available from the corresponding author on reasonable request.
