# Supplementary material for: The effect of blended task-oriented flipped classroom on the core competencies of undergraduate nursing students: a quasi-experimental study
Source: BMC Nurs. 2023 Jan 10;22:1. doi: 10.1186/s12912-022-01080-0 (PMC9830926; doi:10.1186/s12912-022-01080-0)
Supplement: Supplementary file 1 — Additional file 1. [file 12912_2022_1080_MOESM1_ESM.docx]

**Additional file 1：**

**Chapter 2 Section 3 learning task list**

| **Learning content** | **Chapter 2 Section 3**  **Isolation** | | | | | | |
| --- | --- | --- | --- | --- | --- | --- | --- |
| **Learning goals** | Knowledge goals | | Students can correctly explain the concept of isolation, clean area, potentially contaminated area, contaminated area, two channel and buffer room;  Students can state the isolation management and isolation principles;  Students can list common types of isolation and related measures;  Students know the key points of home medical observation in COVID-19 prevention and control;  Students know the specific process of putting on and taking off protective clothing; know the first, second and third level protection standards for COVID-19. | | | | |
|  | Capability goals | | Students can skillfully use caps, masks, goggles, protective masks, decontaminate paper, shoe covers and waterproof aprons, and can skillfully put on and take off isolation clothing. | | | | |
|  | Ideological ethics and professional attitude goals | | Students have the consciousness of scientific research, innovation, self-love and self-improvement; Students have a scientific and rigorous style of work realistic and innovative ideas of craftsmanship spirit of excellence; Students have the concept of caring patients, good professional ethics and professional emotion. | | | | |
| **Tasks** | Preparation before flipped classroom | Contents of self-learning | MOOC | Prevention and control of nosocomial infection (watch the "teaching video", complete the " exercises on knowledge points " and participate in the online discussion in the "Discussion area"). | | Video name. | Video Length (minutes) |
|  |  |  |  |  |  | Isolation and division of work area. | 7.54 |
|  |  |  |  |  |  | isolation management and isolation principles. | 8.0 |
|  |  |  |  |  |  | Types and measures of isolation. | 6.38 |
|  |  |  |  |  |  | Take vital signs of the patient by wearing isolation clothing. | 13.10 |
|  |  |  |  |  |  | Summary of prevention and control of nosocomial infection. | 3.9 |
|  |  |  | Teaching material | Prevention and control of nosocomial infection, isolation ( preview the teaching material and complete the test). | | | |
|  |  | Class task preparation | Group reports | EBOV(Group 4) COVID-19(Group 5) Outbreak and epidemic status, solation types and measures. | | | |
|  |  |  | Group presentation | Wearing and removing Isolation clothing (Group 1). | | | |
|  |  |  | Group homework | Please help the Government of Yemen to develop measures to prevent and control cholera. | | | |
|  |  |  | Pre-test report | Isolation (Content 10 of pre-test report). | | | |
|  |  |  | Extensive learning materials | Intensive reading the paper: "Experience of healthcare-associated infection monitoring from medical team for aiding Hubei Province during COVID-19". | | | |
|  | flipped classroom | **Contents** | | | **Class activities** | | |
|  |  | Isolation and division of work area | | | Group reports:COVID-19 and Ebola、 Q&As、quiz. | | |
|  |  | Isolation management and isolation principles | | | Q&As、group discussion、knowledge extension. | | |
|  |  | Types and measures of isolation | | | Group presentation, group exercise and feedback, relevant literature analysis. | | |
|  |  | Classroom test | | | Quiz at beginning and end of the classroom. | | |
|  | Review after flipped classroom | MOOC | | | Complete the test questions in Section 3 of Chapter 2, read the extensive learning materials, and watch the videos of knowledge points that have not been mastered repeatedly. | | |
|  |  | Learning materials | | | Complete the chapter test of isolation techniques | | |
|  |  | Practice | | | Students make an appointment for laboratory practice in their spare time. | | |
|  |  | Others | | | Search and read other learning literature recommended by the instructor. | | |
| **Learning evaluation** | Self-evaluation of the achievement of learning objectives: all have been achieved, basically achieved, not achieved. | | | | | | |
| **Puzzles and suggestions** | Discuss and communicate with teachers both online (MOOCs discussion area, Cloud teaching materials exchange area, Learning Platform ) and offline (during and after class). | | | | | | |
| Department of Fundamentals of Nursing course, School of Nursing, Hubei University of Medicine | | | | | | | |
